# OptFlux: an open-source software platform for *in silico *metabolic engineering

**DOI:** 10.1186/1752-0509-4-45

**Published:** 2010-04-19

**Authors:** Isabel Rocha, Paulo Maia, Pedro Evangelista, Paulo Vilaça, Simão Soares, José P Pinto, Jens Nielsen, Kiran R Patil, Eugénio C Ferreira, Miguel Rocha

**Affiliations:** 1 IBB-Institute for Biotechnology and Bioengineering/Centre of Biological Engineering, University of Minho, 4710-057 Campus de Gualtar, Braga, Portugal; 2 Department of Informatics/CCTC, University of Minho, Campus de Gualtar, 4710-057 Braga, Portugal; 3 Systems Biology, Dept Chemical and Biological Engineering, Chalmers University of Technology, Kemivägen 10, SE-412 96, Gothenburg, Sweden; 4 Center for Microbial Biotechnology, BioCentrum-DTU, Building 223, Technical University of Denmark, DK-2800 Kgs Lyngby, Denmark

## Abstract

**Background:**

Over the last few years a number of methods have been proposed for the phenotype simulation of microorganisms under different environmental and genetic conditions. These have been used as the basis to support the discovery of successful genetic modifications of the microbial metabolism to address industrial goals. However, the use of these methods has been restricted to bioinformaticians or other expert researchers. The main aim of this work is, therefore, to provide a user-friendly computational tool for Metabolic Engineering applications.

**Results:**

*OptFlux *is an open-source and modular software aimed at being the reference computational application in the field. It is the first tool to incorporate strain optimization tasks, i.e., the identification of Metabolic Engineering targets, using Evolutionary Algorithms/Simulated Annealing metaheuristics or the previously proposed OptKnock algorithm. It also allows the use of stoichiometric metabolic models for (i) phenotype simulation of both wild-type and mutant organisms, using the methods of Flux Balance Analysis, Minimization of Metabolic Adjustment or Regulatory on/off Minimization of Metabolic flux changes, (ii) Metabolic Flux Analysis, computing the admissible flux space given a set of measured fluxes, and (iii) pathway analysis through the calculation of Elementary Flux Modes.

*OptFlux *also contemplates several methods for model simplification and other pre-processing operations aimed at reducing the search space for optimization algorithms.

The software supports importing/exporting to several flat file formats and it is compatible with the SBML standard. *OptFlux *has a visualization module that allows the analysis of the model structure that is compatible with the layout information of *Cell Designer*, allowing the superimposition of simulation results with the model graph.

**Conclusions:**

The *OptFlux *software is freely available, together with documentation and other resources, thus bridging the gap from research in strain optimization algorithms and the final users. It is a valuable platform for researchers in the field that have available a number of useful tools. Its open-source nature invites contributions by all those interested in making their methods available for the community.

Given its plug-in based architecture it can be extended with new functionalities. Currently, several plug-ins are being developed, including network topology analysis tools and the integration with Boolean network based regulatory models.

## Background

Metabolic Engineering (ME) deals with designing organisms with enhanced capabilities regarding the productivities of desired compounds [1]. This field has received increasing attention within the last few years, due to the extraordinary growth in the adoption of white or industrial biotechnological processes for the production of bulk chemicals, pharmaceuticals, food ingredients and enzymes, among other products [2,3].

Many different approaches have been used to aid in ME efforts, taking available models of metabolism together with mathematical tools and/or experimental data to identify metabolic bottlenecks or targets for genetic engineering. Some of these techniques, like Metabolic Control Analysis (MCA), use dynamical representations of the metabolism, while others like Metabolic Flux Analysis (MFA) or Flux Balance Analysis (FBA) apply steady-state stoichiometric models to study the phenotype of microorganisms, under different environmental and genetic conditions (a thorough description of these techniques can be found for example in [1]).

Also based on stoichiometric networks, the field of Pathway Analysis characterizes the complete space of admissible flux distributions, allowing the analysis of the meaningful routes by dissecting them into basic functional units named Elementary Flux Modes (EFMs) [4]. Therefore, EFMs analysis is a valuable tool in ME but its application is limited by two issues: the problem of calculating EFMs in large networks is computationally very hard and, even if this process is successful, their analysis is also difficult, given their high cardinality.

Although many nice examples have been described on successful modifications of the microbial metabolism using the above-mentioned techniques (e.g. some of the examples described in [5]), very few methodologies exist that effectively aid in the rational design of microbial strains by, for example, pinpointing the genetic modifications that can lead to enhanced production capabilities, by using available genome-scale mathematical models (e. g. [6]). This limitation is related with the fact that genome-scale models account for a significant number of genes and reactions, and therefore any resulting ME problem will require quite robust optimization tools.

One of the first approaches to tackle this class of problems was the *OptKnock *algorithm [7], where Mixed Integer Linear Programming (MILP) is used to identify an optimum set of knockouts under a metabolic steady-state approximation. An alternative solution was proposed by the *OptGene *algorithm [8,9], that considers the application of Evolutionary Algorithms (EAs) and Simulated Annealing (SA) in this scenario. These meta-heuristic methods are capable of providing near-optimal solutions within a reasonable computation time, being also quite flexible regarding the objective function that can be optimized (e.g. they are able to deal well with non linear functions).

However, the application of such optimization algorithms and even the use of genome-scale metabolic models for pure simulation has been limited to the developers of the techniques or experienced bioinformaticians, since a platform that provides a user friendly interface to perform such tasks is not yet available. The computation of EFMs is also enabled by some applications, but there is the need of proper tools to conduct the required analysis to fully take advantage on the results in an ME perspective.

Furthermore, the solutions obtained by using those methods or the strategies inferred by model simulations need to be validated before the implementation in the laboratory, because of model uncertainties. This validation is hampered by the complexity of the model itself and of the solutions obtained. In fact, if an ME target is most often not obvious, the analysis of a possible solution given by an algorithm is definitely difficult to interpret and validate.

While, in 2001, the need for mathematical and computational tools to aid in ME efforts was already identified by James Bailey [10], by the time of writing of this text very few user-friendly software tools were still available.

Besides some tools developed a few years ago, such as *FluxAnalyzer *[11] and *MetaFluxNet *[12], recently the *CellNetAnalyzer *[13] (the successor of *FluxAnalyzer*), the COBRA toolbox [14] and the *Systems Biology Research Tool *(SBRT) [15] have been launched. COBRA and *CellNetAnalyzer *are software packages running over the MATLAB environment. Both allow performing many tasks useful in ME like FBA, flux variability analysis and the simulation of gene deletion mutants. *CellNetAnalyzer *is, however, a more comprehensive software tool that allows to analyse metabolic, regulatory and signalling networks. COBRA was built mainly to perform flux and pathway analysis, either with or without experimental data. The SBRT consists of an open-source platform implemented in the Java language and allows performing most of these operations, and also includes other capabilities such as data analysis tools. However, the SBRT and COBRA present an important limitation, since they do not provide a user-friendly interface.

Two other applications have been recently proposed: YANAsquare [16] and SNA [17]. The first is an application developed in Java with a user interface, while the latter is a Mathematica package. Both these tools are essentially focused in calculating the EFMs of a network and using those to perform the analysis of its metabolic capabilities. However, they are quite limited from a ME perspective (although SNA also allows calculating FBA).

Furthermore, none of the aforementioned tools allows to perform strain optimization functions, i.e. they do not *per se *include algorithms for the identification of potential ME targets. Additionally, there is also a need for appropriate model visualization tools associated with simulation software.

Given the huge potential impact of the growing number of genome-scale metabolic models [18], the availability of open source simulation and strain optimization software would be a key to their further development and exploitation. At present, experimenters from both academia and industry find it very difficult to use genome-scale stoichiometric models for simulation and optimization purposes.

Towards the purpose of changing this scenario, we hereby introduce *OptFlux*, a software tool that aims to be the reference platform for the ME community. The main features of this tool are the following:

- Open-source - it allows all users to use the tool freely and invites the contribution of other researchers;

- User-friendly - facilitates its use by users with no/little background in modelling/informatics;

- Modular - facilitates the addition of specific features by computer scientists, given its plug-in based architecture;

- Compatible with standards -compatibility with the Systems Biology Markup Language (SBML) [19] and the layout information of *Cell Designer *[20].

At the current version (2.0), the software accommodates several tools and algorithms that have been developed for the manipulation of metabolic models:

• methods for phenotype simulation, such as FBA, Minimization of Metabolic Adjustment (MOMA) [21] and Regulatory on/off minimization of metabolic flux changes (ROOM) [22];

• methods for MFA, allowing the introduction by the user of values for experimentally measured fluxes and calculating the effects on the flux space;

• Elementary modes analysis, allowing the calculation of the set of EFMs for the network and its visualization and further analysis;

• strain optimization algorithms: OptKnock, EAs and SA.

• a suitable model visualization tool to facilitate the interpretation of results.

To the best of our knowledge, this is the first tool that allows performing strain optimization in a user-friendly interface and the first effort to create a community-based and community-oriented software for ME with such characteristics.

The main concepts used in the development of *OptFlux *and its main functionalities are presented in the next sections.

## Implementation

*OptFlux *is fully implemented in the Java language, which is being increasingly used by the scientific community. *BioVisualizer *is based on the *Jung *Java library [23]. The only non-Java parts consisted on the *GNU Linear Programming Kit *(GLPK) [24] used to execute all linear programming and MILP computations and the *LibSBML *[25] used to handle files in the SBML format.

To ensure modularity, *OptFlux *is implemented in such a way that new features and services are easily plugged in. It is entirely built on top of *AIBench *[26], a software development framework that was born as a collaborative project between the authors and researchers from the University of Vigo in Spain.

Building applications over AIBench brings important advantages to both the developers and the users, given its design principles and architecture. The applications incorporate the three types of well defined objects described before: operations, *datatypes *and *datatype views*, following the MVC (model-view-controller) design pattern. This leads to units of work with high coherence that can easily be combined and reused. Furthermore, it is plug-in based: applications are developed adding components, called plug-ins, each containing a set of *AIBench *objects. This allows reusing and integrating functionality of past and future developments based on *AIBench*.

## Results

**Figure 1:**
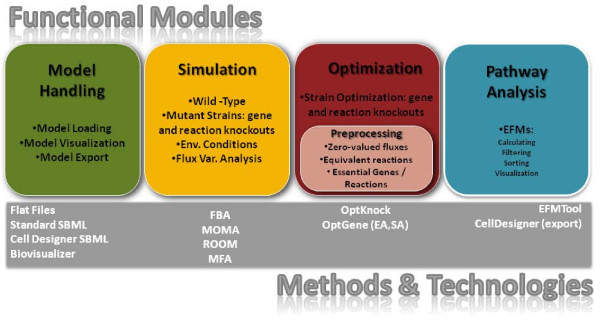
**Functional modules of the *OptFlux *application**.

**Figure 2:**
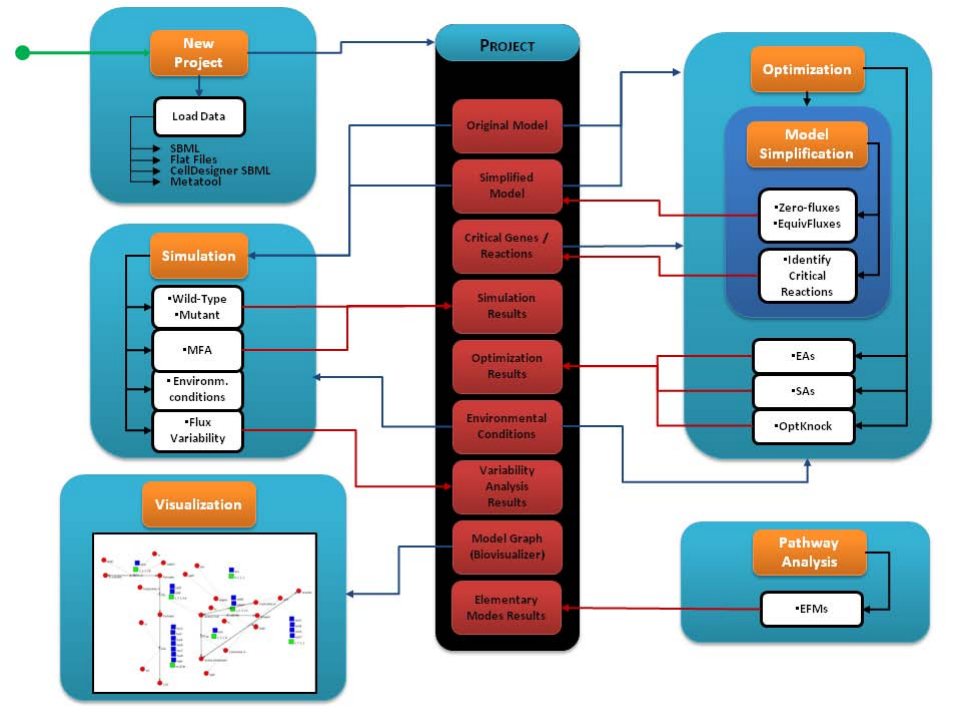
**Main functionalities and fluxes of information in the *OptFlux *application**.

*OptFlux*'s main capabilities can be grouped into distinct functional areas that will be described in detail below. Figure 1 shows the high-level organization of *OptFlux*, including the main operations that can be performed within the software. In Figure 2, a schematic representation of the main functionalities of *OptFlux *is given, showing the typical fluxes of information. Starting with a stoichiometric metabolic model that can be loaded in different formats (SBML, Metatool or flat files), the user can perform simulations under different environmental and genetic conditions (using either FBA, MOMA or ROOM), investigate ME targets for improving the production of desired compounds, analyze the flux space given a set of measured fluxes with MFA methods or perform the computation and further analysis of the EFMs.

The full description of the currently implemented features is provided in the application's set of How To's available at the project's website. Furthermore, a Beginner's tutorial is available for helping first-time users.

### Model handling

Regarding model handling, *OptFlux *makes available a number of operations to visualize, import and export stoichiometric metabolic models, including reactions, metabolites, equations and, if available, gene-reaction associations. It allows the loading of models either from flat text files (containing the lists of reactions, metabolites, the stoichiometric matrix and gene-reaction associations), from text files following the Metatool [27] format or from files complying with the SBML standard. The compatibility with SBML allows the use of models stored in public databases, e. g. *BioModels *[28] or the *BiGG *database [29] or built using other software tools, e.g. *Cell Designer *[20]. The process of loading a model is facilitated by the development of a wizard that encompasses several steps, where the user can choose from a number of options related to the format of the input files.

External metabolites and biomass formation reactions are automatically identified from the input files based on an explicit definition, compartment information or by patterns in the names. This information can then be validated or edited by the user.

### Simulation

The Simulation area encompasses the metabolic phenotype simulation methods implemented in *OptFlux*, i.e. the algorithms that calculate the values for the fluxes over the whole set of reactions in the model. It is possible to perform simulations of the wild-type (see note 1) and mutant strains. In the first case, the original model is considered with no additional constraints, while in the latter a number of user selected reactions (or genes if the model includes gene-reaction associations) are removed from the original model before simulation. The simulation results include, besides the flux values, net conversions and shadow price information and are placed in *OptFlux*'s clipboard area, becoming easily accessible for further analysis or future operations.

One other feature available is the ability to define specific *Environmental Conditions*. These are created by selecting a set of drain reactions (reactions that stand for the intake and secretion of external metabolites) and, then, imposing constraints over the values of their fluxes. As an illustrative example, this allows the definition of aerobic or anaerobic conditions by changing the limits in the oxygen uptake reaction. Environmental conditions can be used in both wild-type and mutant simulations.

*OptFlux *has three available methods for conducting the simulations: FBA (see for example [30]), MOMA [21] or ROOM [22]. The first method uses a Linear Programming (LP) formulation to calculate the values of all the fluxes over the reactions and can be used to simulate either wild-type or mutant strains. To reach the FBA solution, by default the flux over the reaction that represents biomass formation is the one being maximized, since this has proven to be a good representation of the natural behaviour of microorganisms in many circumstances [31], but it is possible to perform simulations by maximizing or minimizing any flux of the model.

MOMA and ROOM are appropriate only for the simulation of mutants, since they calculate the minimum distance solution or the solution with minimum number of changes, respectively, for the mutant strain relative to the original "wild-type" solution (i. e. obtained with FBA). MOMA uses a Quadratic Programming formulation while ROOM is implemented based both on the original MILP formulation and an LP relaxation of the original MILP problem (proposed by the original authors) [22].

*OptFlux *also includes some features for Flux Variability Analysis (FVA). Currently, there are two tools available, allowing to:

• Calculate the maximum possible value of a selected flux, for a range of fixed values for the biomass flux (typically varying from 0 to 100% of its value in the wild-type strain);

• Calculate the maximum and minimum limits for all fluxes in the model, given a constraint imposed by a user-defined minimum biomass value. If this value is zero, this is equivalent to compute the tight bounds of the fluxes for all reactions.

The calculation of the fluxes can also be performed adding experimental data, used to constrain the original metabolic model, using MFA methods. Depending on the number of measured fluxes, the resulting system can be classified as underdetermined, determined or over-determined. Determined and over-determined systems are solved using the methods described in [1]. Concerning underdetermined systems, there are no unique solutions for the unknown flux set. Thus, an FBA problem is formulated and solved as described previously. Furthermore, it is possible to compute the tight bounds respecting the measured constraints.

### Optimization

The strain optimization area provides the users with interfaces to identify sets of reaction deletions (or gene deletions if gene-reaction associations are available) that maximize a given objective function related with a desired industrial objective. The ultimate purpose of the implemented algorithms is to identify genetic modifications that force the microorganism to produce a particular metabolite, while still obeying the physiological aim of maximizing biomass production.

The OptKnock algorithm [7] and two meta-heuristic optimization methods, EAs and SA [8], are currently available. The first was implemented following the original formulation [7] and also the methods described in [32]. It should be noted that in our implementation only freely available solvers can be used, while in previous work the commercial CPLEX solver has been used. Also, from our experiments, running OptKnock in genome-scale models (such as the one from our case study) is quite demanding and can lead to situations of numerical instability in the solver.

On the other hand, the metaheuristics are configured with some default parameters, using set-based representations that can search through fixed-size or variable-size solutions. In the first case, the user specifies the number of allowed reaction/gene deletions, while in the latter the optimization algorithm also performs the automatic discovery of the optimum value for that variable. Both methods were implemented by the authors and the results in several case studies have been previously presented [8].

At present, *OptFlux *allows to maximize two different objective functions: Yield and Biomass-Product Coupled Yield. In the first case, the yield on the desired compound is targeted but a minimum biomass level is imposed, while the second searches for mutants that are likely to exhibit higher productivities, since biomass production is also included in the objective [8].

The high number of variables typically involved in a genome-scale metabolic model makes the optimization task computationally hard. Thus, it is important to be able to simplify the models without compromising their accuracy and information content. In this context, two alternatives are available: to simplify the model in terms of its structure (these operations are valid in every scenario, i.e. considering all environmental conditions) and also to simplify the model using simulation, calculating the limits of the reaction fluxes using a simulation method such as FBA.

In the structural simplification context, two operations are available: finding zero valued reactions, i.e. reactions that are mathematically constrained to have the value of zero for the corresponding flux and, also, finding equivalent reactions, i.e. reactions that are constrained to have the same flux value and, therefore, can be replaced by a single reaction.

Regarding the simulation-based simplification operations, the original bounds can be replaced by the calculated limits. Also, this method can be used to identify zero valued fluxes (reactions for which both new lower and upper limits of the fluxes are zero). It is important to notice that this method is dependent on the environmental conditions defined.

Another feature provided is an automatic method for the discovery of essential reactions, i.e. reactions that when disabled, make the organism non-viable. If gene-reaction associations are included in the model, a similar operation can be defined for the discovery of essential genes. In both cases, an organism is found to be viable if the value for the biomass flux is significantly larger than 0 (i.e. larger than 5% of the wild type value). The essential genes or reactions are not used as targets for optimization, since they would unnecessarily increase the number of decision variables and therefore the size of the search space.

### Elementary flux modes analysis

Optflux also allows state-of-the-art EFM calculation provided by the *EFMTool *[33] that implements one of the most efficient algorithms available. Moreover, it provides a simple user interface that allows an intuitive filtering of the results that match given patterns.

After the computation of the EFMs, the net conversion associated with each EFM is calculated (only unique conversions are maintained). Furthermore, for each net conversion, the greatest common divisor is calculated to improve the reading of the conversion equation. To do so, all the coefficients have to be integers and, therefore, the EFM calculation is limited to using big integer arithmetic.

In the filtering step, EFMs can be selected based on the presence/absence of external metabolites in the net conversions. Moreover, they can also be sorted by yield, assuming that an input and an output metabolite are provided.

The user can browse through the filtered conversions in a table that presents the conversion equation, yields and provides access to the set of related EFMs. This viewer also allows row sorting based on any column criteria. The visualization of the EFMs is presented in a column-wise table, where each column corresponds to an EFM and each line to a reaction of the model. Each EFM, i.e. its flux values, can be exported to *Cell Designer*, if the model was created from a *Cell Designer *SBML file. For each reaction in the EFM, the line in the *Cell Designer *layout is represented with a thickness that is proportional to the value of the flux.

### Visualization

*OptFlux *allows the graphical visualization of the pathways through *BioVisualizer*, a visualization plug-in that was developed to represent metabolic networks as graphs, with a number of distinct node types (e.g. metabolites, enzymes, reactions) and connections.

If a *Cell Designer *SBML file is loaded as the model source, automatically it will be used by *BioVisualizer *in the visualization operations, using the layout built previously in *Cell Designer*. Also, if the original model is loaded from flat files or normal SBML, *BioVisualizer *can work if a *Cell Designer *SBML file is available, typically representing only a subset of the whole model (e.g. a pathway) with compatible names for the reactions.

One of the major features of this tool is the ability to associate numerical values to the different types of nodes and edges. This allows the visualization of the metabolic network overlapped by the values of the fluxes obtained in a given simulation. Moreover, the flux values can be exported to *Cell Designer *if the model was created from a *Cell Designer *SBML file.

### User interaction

*OptFlux *development has taken as a first premise to build a tool aimed at biotechnology researchers and not at computational or bioinformatics experts. Thus, the primary goal in the development process was to provide good usability, valuing the simplicity and intuitiveness of the tool.

The user interaction is based on three main concepts, used throughout the application:

• *Datatypes*: represent the distinct types of objects holding the relevant data to the application (such as models, simulation or optimization results, etc). Each type can have multiple instances (objects) within the application.

• *Views*: represent different ways to visualize the contents of data objects. Each datatype can have one or more methods to visualize its instances.

• *Operations*: represent the software functionalities or available actions. When an operation is called, its interface is launched and the input data objects are selected. After being triggered, an operation typically changes or creates an instance of the output datatype.

**Figure 3:**
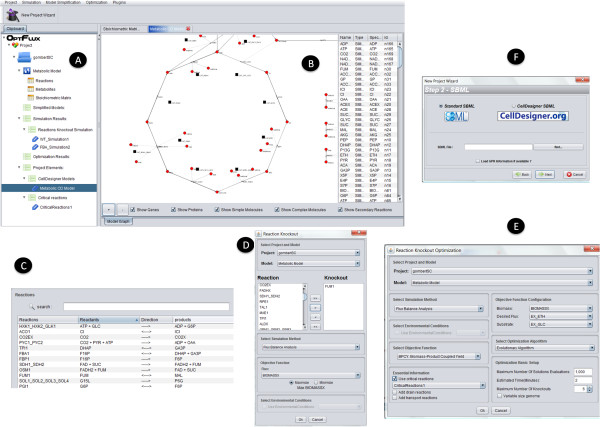
**Screenshots of *OptFlux***: a) Clipboard containing the main datatypes; b) view of model graphical representation; c) one of the available views of the stoichiometric model; d) mutant simulation operation; e) Optimization with EA's; f) wizard for starting a new project.

Based on these concepts, a user-friendly Graphical User Interface (GUI) was developed. The original layout of the components can be observed in the screenshots presented in Figure 3.

The clipboard on the left (Figure 3a) keeps all data objects created within the application, in a logical hierarchy, grouped by their *datatypes*. The root of this tree is the *Project datatype *that keeps all objects related to a given metabolic model and the analysis performed with it.

The components of a project are graphically shown in the form of explicit hierarchical containers, namely:

• The metabolic model, including the sets of metabolites (internal and external), the set of reactions with their flux bounds and stoichiometry, the steady state equations and, when available, the encoding genes and the gene-reaction association rules;

• A set of simplified models that are the result of model simplification operations;

• Sets of simulation and optimization results, including also MFA and flux variability analysis results;

• Other optional objects grouped in the project elements list, including: a model graph for visualization to be used by *BioVisualizer*, environmental conditions, lists of essential genes/reactions, results from EFMs computation, among others.

When an object in the clipboard is double-clicked, the views corresponding to its *datatype *will be launched on the right side of the working area (if more than one view is available, those are accessible in different tabs). Examples of two views of a metabolic model are shown in Figures 3b and 3c.

All the available operations are easily accessible, either through the menu in the top or by right clicking the item in the clipboard area, an action that displays all operations that work over that type of argument. Snapshots of simulation and optimization operations are shown in Figures 3d and 3e.

To make the usage of the software easier, a wizard was developed for creating a new model (Figure 3f). This wizard is visible in the toolbar and is also available in the menu. It encompasses a number of steps that allow the user to define the setup for each operation in a straightforward way.

All operations are, at the maximum possible level, default-oriented, thus hiding behind scenes their complexity (e.g. definition of non-obvious parameters). Nevertheless, they allow more advanced users to fine-tune the parameters available to a given operation.

### Usage example: succinate production with *E. coli*

To illustrate the main features of the application, a case study is shown here that considers the microorganism *Escherichia coli *and where the aim is to produce succinic acid, with glucose as the carbon source. The genome-scale model used in the simulations was developed by [34], considering the whole *E. coli *metabolic network with a total of 1075 fluxes and 761 metabolites. A simpler example is given in the Tutorial (available at the website) where a small model of *Sacharomyces cerevisiae *is used.

Succinic acid is one of the key intermediates in cellular metabolism and therefore an important case study for ME strategies [35]. In fact, knockout solutions that lead to improved phenotypes regarding the production of succinic acid are not straightforward to identify since they involve a considerable number of interacting reactions. Additionally, it is a chemical used as feedstock for the synthesis of a wide range of other chemicals with several industrial applications (e.g. food industry). Currently, it is mainly produced through petrochemical processes that can be expensive and have significant environmental impacts. *E. coli *has many advantageous characteristics as a production host, such as rapid growth under aerobic and anaerobic conditions and simple nutritional requirements.

**Figure 4:**
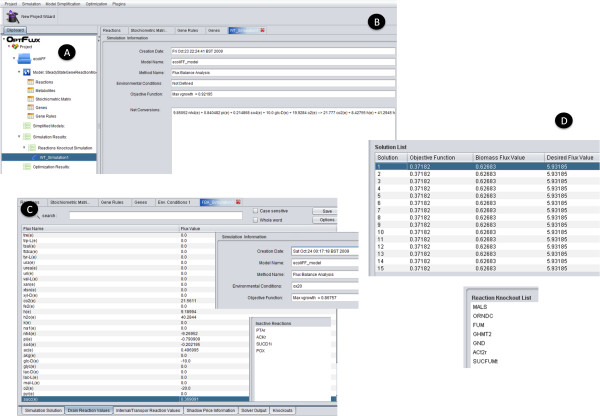
**Screenshots of the *E. coli *case-study**: a) clipboard containing the original metabolic model, simplified model, simulation results and optimization results; b) view of the simulation results (objective function and net conversions) obtained for the wild-type strain; c) view of the simulation results (FBA) obtained for a mutant with enhanced capabilities regarding succinate production; d) view of the results obtained for the optimization using EAs and knockout list for the best solution found;

In this case study, the main steps depicted in Figure 4 were followed and are described next (all the referred files are available at the project's web site):

- The model was created using the fluxes, metabolites, stoichiometric matrix and gene-reaction rules flat files. A *Cell Designer *SBML file is loaded afterwards, for visualization purposes, representing the Pyruvate metabolism pathway.

- A simulation of the wild type strain is performed, using FBA and maximizing biomass (the vgrowth flux). This results in a flux of 0.92 gDW/gDW/hr (or simply hr^-1^) for the biomass (glucose uptake rate fixed at 10 mmol/gDW/hr) (Figure 4b). The wild-type strain exhibited no production of succinate, as expected.

- A simulation of a mutant strain with four knockouts known to produce succinate [36] was performed and the excretion of 0.37 mmol/gDW/hr of succinate was obtained (Figure 4c). The mutant strain exhibits a growth rate that is 94% of the wild-type. This strain was simulated constraining the oxygen uptake flux to 20 mmol/gDW/hr.

- A Simulated Annealing algorithm was used for identifying additional mutants with succinate production capabilities, with BPCY as the objective function. The best results obtained are shown in Figure 4d, where it is clear the great improvement in the secretion of succinate for all the solutions found, as compared with the published strain. The best succinate yield obtained was 0.593 mol succinate/mol glucose, for a strain that exhibits a growth rate that is around 68% of the wild-type and with 8 reactions removed. However, additional solutions have been found showing the ability of the selected optimization algorithms and the chosen objective function to provide a family of near-optimal solutions.

In a real case, the following steps would be the examination and comparison of the mutant strains obtained *in silico *before the laboratory implementation of the pinpointed knockouts.

Other distinct examples can be used to illustrate the implementations of the features of MFA, EFMs and OptKnock. Those are all available in the project's web site (in the How To's sections).

## Conclusions

The *OptFlux *software is, to the best of the authors' knowledge, the first freely available computational tool for *in silico *ME that supports the set of described methods. Still, there are some tools available that are able to perform some of the tasks mentioned above. From all the surveyed software tools, the *CellNetAnalyzer *[13], the COBRA toolbox [14] and the *Systems Biology Research Tool *(SBRT) [15] are the most similar to *OptFlux*, in terms of the available features. In Table 1, a comparison of the four tools (including OpfFlux) is performed, listing the features available in each application. In all cases, simulation with FBA and support to basic standards is included. However, only *OptFlux *is able to perform strain optimization, being the first ME computational tool to provide algorithms to reach ME targets given a user-defined objective function and working with genome-scale models.

**Table 1:** Feature comparison of several tools for metabolic network analysis.

		Other tools		
	
	OptFlux	*CellNetAnalyzer*	COBRA	SBRT
**File formats/standards**				

- SBML	•	•	•	•

- Metatool format	•	•		

- Flat files	•	•	•	•

**Phenotype simulation**				

- FBA: wild type, environmental conditions, gene deletion mutants	•	•	•	•

- Dynamic FBA			•	

- ROOM	•			

- MOMA	•		(1)	

- MFA basic methods	•	•		

- Gene-reaction associations	•		•	

- Regulatory network simulation	(2)	•		

**Strain optimization**				

- OptKnock algorithm	•			

- Metaheuristics: OptGene	•			

**Metabolic Network Analysis**				

- Elementary Flux Modes	•	•		

- Minimal cut sets		•		

- Flux Variability Analysis (FVA)	•	•	•	•

- Topological network analysis	(2)	•		(3)

**Visualization**				

- Built-in visualization	•	•		

- Interaction with CellDesigner	•			

- Interaction with Cytoscape		•		

**Other features**				

- Graphical user interface	•	•		

- Does not depend on commercial software	•			•

- User documentation	•	•	•	•

Notes

(1) Implementation based on linear programming formulation (linear MOMA)

(2) Plug-ins under development

(3) Includes some basic graph analysis methods

The table provides a comparison of the main features of OptFlux with the major alternatives in metabolic network analysis. Features have divided into six groups: file formats and standards, phenotype simulation, strain optimization, network analysis, visualization and other features.

Furthermore, *CellNetAnalyzer *and COBRA are based on a commercial platform (MATLAB) and the latter does not supply any kind of user-friendly interface. The SBRT consists on an open-source platform implemented in the Java language, but it provides only a basic GUI, merely to launch the execution of its processes. We can, therefore, conclude that *OptFlux *provides the first freely available ME workbench that addresses the needs of biotechnologists, providing a user-friendly environment.

The remaining efforts in this field are quite distinct from *OptFlux *so we will not refer to their features in detail. It should be mentioned that efforts such as *Cytoscape *[37], *Cell Designer *[20] and *Systems Biology Workbench *[38] are considered by the authors as very important projects, although orthogonal to our work, and ways to integrate *OptFlux *with those tools are being considered.

Since *OptFlux *aims at being the reference software tool for the ME community, there are some near-future plans for the implementation of additional features useful for the analysis and manipulation of metabolic models. These are being developed as new plug-ins, to facilitate their integration as modules that users can optionally install to enlarge the functionalities of the workbench. The main current efforts are focused on the development of the following plug-ins:

• Topological analysis of metabolic networks;

• Integration of Boolean network-based regulatory models with the existing metabolic models, allowing for phenotype simulation and strain optimization;

• Strain optimization approaches based on multi-objective EAs.

Also, the connection to relevant databases such as *KEGG *[39] or *BioCyc *[40] is a path worth exploring. Currently, *OptFlux *has a plug-in that supports link-outs, i.e. it allows users to automatically launch searches over relevant databases from the names of entities (reactions, metabolites, genes) present within the viewers of the metabolic models. The databases used in each case can be configured in the setup of the plug-in.

## Availability and Requirements

The software is made available, together with other resources, in the home page given below.

More details:

- Software name: OptFlux - software for metabolic engineering

- Project home page: http://www.optflux.org

- Operating system(s): Platform independent

- Programming languages: Java

- Other requirements: Java JRE 1.6.x, GLPK

- License: GNU-GPL, version 3

## Abbreviations

BPCY: Biomass-Product Coupled Yield; DW: Dry Weight; EAs: Evolutionary Algorithms; EFMs: Elementary Flux Modes; FBA: Flux Balance Analysis; FVA: Flux Variability Analysis; GLPK: GNU Linear Programming Kit; LP: Linear Programming; ME: Metabolic Engineering; MFA: Metabolic Flux Analysis; MILP: Mixed Integer Linear Programming; MOMA: Minimization of Metabolic Adjustment; MVC: Model View Controller; ROOM: Regulatory on/off minimization of metabolic flux changes; SA: Simulated Annealing; SBML: Systems Biology Markup Language; SBRT: Systems Biology Research Tool.

Note 1: Definition of "Wild-type" strains in the context of OptFlux simulations

We use the term wild-type strain/organism in this study meaning strains that have a (approximately) known pre-definable steady-state flux objective function (e.g. biomass formation rate as in case of FBA). This terminology is used solely for the purpose of intuitive understanding of the methods and tools and therefore it should not be interpreted in the biological sense of wild-type strains/organisms.

## Authors' contributions

IR, MR, PM, KRP were involved in the conception of the algorithms. IR, MR, PM, PE, PV, SS and JPP were involved in the design and implementation of the software. IR, KRP, ECF and JN proposed and prepared the case study. IR, MR, PM and KRP helped to draft the manuscript. All authors read, reviewed and approved the final manuscript.

## Acknowledgements

The authors wish to thank the financial support of the following institutions:

- the company Dupont under the scope of the "Dupont European University Support Program Award";

- The European Commission under the scope of the European Coordination Action SYSINBIO - Systems Biology as a Driver for Industrial Biotechnology;

- The project MIT-PT/BS-BB/0082/2008 sponsored by Portuguese FCT;

- The Luso-American Development Foundation under the scope of the Computational Biology Collaboratorium.
